# Habenular Stimulation for Neurosurgery Resistant Obsessive-Compulsive Disorder: A Case Report

**DOI:** 10.3389/fpsyt.2020.00029

**Published:** 2020-02-07

**Authors:** Chencheng Zhang, Yingying Zhang, Dianyou Li, Zhengdao Deng, Bart Nuttin, Valerie Voon, Bomin Sun

**Affiliations:** ^1^ Department of Functional Neurosurgery, Ruijin Hospital, Shanghai Jiaotong University School of Medicine, Shanghai, China; ^2^ Department of Neurosurgery, University Hospitals Leuven, Leuven, Belgium; ^3^ Department of Psychiatry, University of Cambridge, Cambridge, United Kingdom

**Keywords:** habenular, OCD (obsessive-compulsive disorder), brain stimulation, deep brain simulation, YBOCS = Yale-Brown Obsessive Compulsive Scale

## Abstract

**Background:**

Some patients suffer from persistent and severely disabling Obsessive-Compulsive Disorder (OCD) symptoms that cannot be alleviated by conventional treatments or neuroablative interventions targeting cortico-striatal loop circuits. Currently, it is unclear how to manage the clinical symptoms of these unique patients. We reasoned that deep brain stimulation (DBS) of the habenula (HB) could be a valuable subsequent treatment option for these otherwise medically intractable cases of severe OCD. The HB is an epithalamic structure critically involved in the encoding and responding to aversive stimulus events, cognitive and brain processes known to be impaired in many patients with OCD. Similarly, HB DBS can alleviate depression and anxiety, which often co-occur with OCD. Here, we explore the clinical benefits and risks of HB DBS treatment in a patient with severe and refractory OCD.

**Case Presentation:**

A 30-year-old male patient presented with persistent and severely disabling OCD symptoms that were refractory to previous psychological and pharmacological treatments as well as to neuroablative surgical interventions involving both capsulotomy and cingulotomy. After HB DBS, however, the severity of the patient's OCD symptoms was markedly reduced at 1-month follow-up, which was sustained until the final (at 12-month) follow-up. The patient also reported enduring improvements in depression, anxiety, and health-related quality of life after several months of HB DBS treatment.

**Conclusions:**

This case report provides the first clinical evidence suggesting that HB DBS could serve as a safe and effective alternative neurosurgical intervention for severe and refractory OCD. The present findings are promising and warrant further research into the role of the HB in pathophysiology and treatment of OCD.

## Background

OCD is a psychiatric disorder with a lifetime prevalence of 1–3%. Pharmacotherapy and psychological intervention, particularly cognitive behavioral therapy (CBT), are the first-line therapies for OCD, achieving an overall response rate of 40–60%. Among the non-responders, about 10% is characterized by persistent and severe clinical symptoms, which profoundly impair the patient's functioning and quality of life. As a result, neurosurgical interventions, particular ablative surgery and deep brain stimulation (DSB), have been employed in an effort to manage the severely disabling and treatment-refractory OCD symptoms in this subset of patients. Although the pathophysiology of OCD remains incompletely understood, preclinical and clinical studies have implicated the involvement of abnormal functioning in medial and orbital frontal-basal ganglia–thalamic circuits. In line with this hypothesis, stereotactic neurosurgical lesions made in the anterior limb of the internal capsule, the anterior cingulate, or the subcaudate region, lesions that all affect the key circuits implicated in OCD pathophysiology, can improve severe OCD symptoms in patients who are refractory to conventional treatments. However, ablative neurosurgical interventions are not reversible and have traditionally been considered as a last resort intervention for severe and refractory OCD. Moreover, stereotactic ablative neurosurgical interventions are not effective for all patients, yielding a response rate of 50–60% (1). Correspondingly, it is currently unclear how to manage patients with severely disabling OCD symptoms who have also failed to respond to stereotactic ablative surgery (2).

We reasoned that DBS of the habenula (HB) could offer a promising subsequent treatment option for these otherwise medically intractable cases of severe OCD. The HB is an epithalamic structure that serves as an important link between forebrain and midbrain regions. This structure has recently received much clinical interest because it modulates both dopaminergic and serotonergic neurotransmission (3). The lateral HB is believed to play a crucial role in negative reward processing, particularly the anticipation of aversive sensory stimuli (via input from basal ganglia) and the behavioral responses to stress, pain, fear-provoking or other aversive stimulus events (via input from the limbic system), ultimately functioning to suppress motor activities that lead to an aversive outcome (4). Because OCD is associated with core deficits in stimulus avoidance learning and behavioral control (5), we hypothesized that DBS of the HB could be a novel and effective subsequent treatment option for those patients who have failed to respond to stereotactic ablative surgery. Moreover, several studies have reported that HB DBS treatment can alleviate affective disturbances in patients with refractory mood disorders (6, 7). Therefore, HB DBS could serve to improve not only the symptoms of OCD but also associated cognitive deficits, depression, anxiety, and quality of life. Here, we describe an adult male patient who had failed to respond to ablative surgery but subsequently showed widespread clinical improvements over one year of HB DBS treatment.

### Case Presentation

At the time of HB DBS surgery, the patient was 30 years of age and had suffered from severe OCD (according to DSM-IV criteria) since the age of 15 (**Table 1**). His primary symptoms consisted of both obsessions and compulsions, including intrusive negative thoughts, excessive cleaning behaviors, and “just right” feelings. He had tried but failed to respond to evidence-based psychological treatments, including cognitive behavioral therapy. Similarly, his OCD symptoms had failed to respond to multiple trials of pharmacotherapy of adequate duration and dose, including augmenting medications (**Table 1**). In 2012, the patient underwent bilateral anterior capsulotomy, but this neurosurgical intervention had limited therapeutic effects; the patient selection criteria used for this surgical intervention has been described elsewhere (8). In 2014, the patients received bilateral cingulotomy, which was also mostly ineffective. Subsequently, the patient was offered HB DBS after he had requested us to consider a potential further treatment for his severely disabling OCD symptoms. The patient was then enrolled in a study on HB DBS treatment for refractory OCD. This study was approved by the Ruijin Hospital Ethics Committee of Shanghai JiaoTong University School of Medicine. Written informed consent was obtained for study participation and the publication of this case report. Bilateral HB DBS electrodes (L301, PINS, Beijing) were implanted under general anesthesia. The surgical procedure used to target the HB and implant the electrodes has been described in another study (6).

**Table 1:** Patient's demographic and clinical characteristics.

Gender	Male
**Age**	30
**Marriage**	Married
**Occupation**	Unemployed
**Illness Duration**	15 years
**Hospitalization times**	6
**Drug Trials**	Fluvoxamine, Fluoxetine, Citalopram, Clomipramine, Olanzapine, Magnesium Valproate, Trihexyphenidyl
**Drug Usage Pre-operation**	Fluvoxamine (100mg/d); Benzhexol (2mg/d); Olanzapine(7.5mg/d); Magnesium Valproate (0.25g/d)

**Figure 1:**
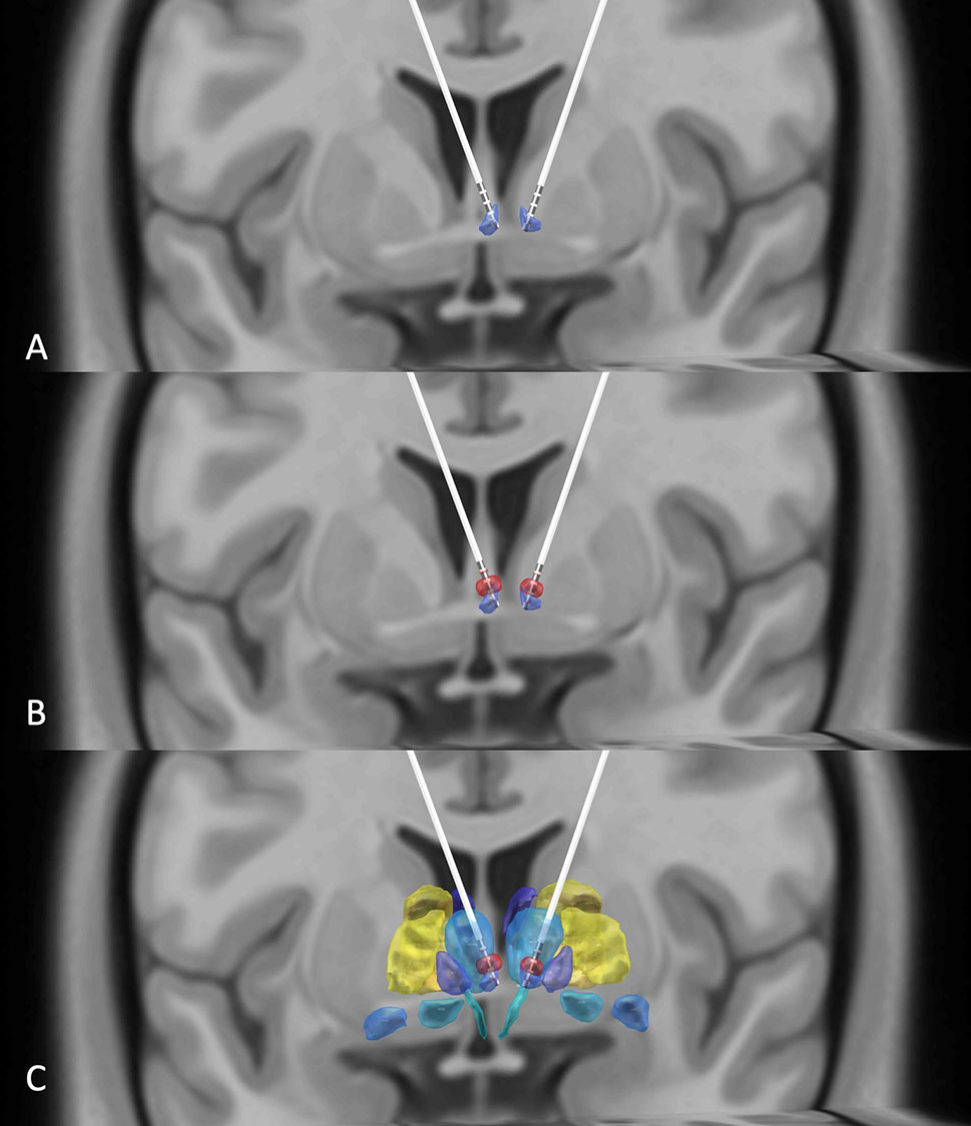
**(A)** DBS electrodes placed in the habenula region (purple) using the Thomas Atlas (Saranathan, 2019) as visualized by reconstructing preoperative T1, T2, and postoperative CT images. **(B)** Illustration that the volumes of activated tissues (red) induced by the DBS electrodes overlap with the patient's habenula nuclei. **(C)** A view of the habenula (purple), volume of activated tissues (red), and the adjacent thalamic regions (blue) using the Thomas Atlas (9).

On the same day of the HB DBS surgery, the location of the electrodes implanted in the HB was confirmed and the HB DBS treatment initiated. Clinical outcome assessments were repeatedly performed over the study follow-period of 12 months by an experienced rater who was not blinded to the patient's condition and HB DBS treatment. The patient's dosage of medications before surgery was kept fixed during the study follow-up. After DBS parameter optimization (6), the final DBS parameters used were: left: C+2-, 1.6 V, 60 μs, 60 Hz; right: C+2-, 1.35 V, 60 μs, 60 Hz. The coordinates of active contacts were X = 89.7, Y = 99.7, Z = 96.7; X = 100, Y = 99.9, Z = 95.7 (**Figure 1**). We utilized a relatively low voltage and pulse width to lower the risk of adverse side effects in this patient, such as headache and dizziness. The side effects of different stimulation threshold values are listed in **Table 2**.

**Table 2:** Adverse side effects of different stimulation threshold values.

	Right Habenula				Left Habenula			
**Contact**	1-	2-	3-	4-	1-	2-	3-	4-
**Voltage/V**	2	1	1	1	1	1	3	3
**Side Effect**	Dizzy	Dizzy Numb Nausea	Dizzy	Dizzy Nausea	Dizzy Flustered	Dizzy	Dizzy Flustered	Dizzy Nausea Numb

Case+, 60 μs, 60 Hz.

After the onset of HB DBS, the severity of the patient's OCD symptoms was markedly reduced at 1-month follow-up (**Table 3**). The improvements in both his obsessions and compulsions were maintained over the course of the entire follow-up period. At 12-month follow-up, the patient's overall level of OCD symptoms, as measured by using the total score on the Yale-Brown Obsessive-Compulsive Scale (YBOCS-II) (10), was reduced by about 35% as compared to his total score obtained at baseline (**Table 3**). After HB DBS onset, the patient also reported improvements in depression, anxiety, and health-related quality of life at 3-month follow-up, which were sustained until 12-month follow-up (**Table 3**). The patient's sleep quality was not affected consistently by the HB DBS treatment.

**Table 3:** Clinical assessment before and after deep brain stimulation surgery.

Rating Scale	Baseline	1 m	1.5 m	3 m	4.5m	6 m	7.5 m	12 m
**YBOCSII**	31	21	25	19	21	22	25	20
Obsession	19	11	15	10	12	13	15	12
Compulsion	12	10	10	9	9	9	10	8
**HDRS**	11		6	8		5	9	7
**HARS**	13		11	8		7	8	9
**PSQI**	5			3		7	5	6
**EQ-5D-5L**								
Mobility	2			1		1	1	1
Self-care	1			1		1	1	1
Usual activity	1			1		1	1	1
Pain/Discomfort	2			1		1	1	1
Anxiety/Depression	2			2		2	1	2
Health today	60			80		90	95	80

YBOCSII, Yale-Brown Obsessive-Compulsive Scale, Second Edition;

HDRS, 17-item Hamilton Depression Rating Scale;

HARS, Hamilton Anxiety Rating Scale;

PSQI, Pittsburgh sleep quality index;

EQ-5D-5L, EuroQol-5 Dimension-5 Level;

m, Month(s).

### Discussion and Conclusion

We utilized HB DBS in an effort to manage chronic and severely disabling OCD symptoms in a unique patient who had failed to respond not only to conventional treatments but also to stereotactic ablative neurosurgery (capsulotomy and cingulotomy). After HB DBS onset, the patient showed a clinically significant improvement in both his obsessions and compulsions at 1-month follow-up, which was maintained over the 12-month treatment course. Moreover, he experienced enduring improvements in depression, anxiety, and health-related quality of life after 3 months of HB DBS treatment. Although we cannot rule out placebo effects, this possibility does not seem likely, given the patient's long disease duration and history of multiple treatment failures while showing a sustained clinical response to HB DBS in this study.

Finally, the HB DBS was well tolerated by the patient, and no significant adverse events occurred during the surgery and follow-up period.

Based on these results, we tentatively conclude that the HB could be a new therapeutic target that is distinct from the cortico-striatal loop circuits traditionally targeted in prior neurosurgical interventions for severe and refractory cases of OCD. If this preliminary conclusion turns out to be valid, HB dysfunction would play an important role in the pathophysiology of OCD, too. The implication is that OCD pathophysiology could be multi-focal or more widespread than previously thought (2), involving not only abnormal dopaminergic neurotransmission in the striatum but also abnormal serotonergic function modulated by the HB. To our knowledge, this case report provides the first clinical evidence suggesting that HB DBS may be a safe and effective intervention for severe and refractory OCD. Our observations seem promising enough to warrant further research into the role of the HB in pathophysiology and treatment of OCD.

## Data Availability Statement

The datasets generated for this study are available on request to the corresponding author.

## Ethics Statement

The studies involving human participants were reviewed and approved by Ruijin Hospital, Shanghai Jiaotong University School of Medicine. The patients/participants provided their written informed consent to participate in this study.

## Author Contributions

Design: CZ, DL, BS. Surgery: DL, BS. Assessment: YZ, VV. Write: CZ, ZD, BN.

## Conflict of Interest

The authors declare that the research was conducted in the absence of any commercial or financial relationships that could be construed as a potential conflict of interest.
